# Potential Role of miRNAs in Developmental Haemostasis

**DOI:** 10.1371/journal.pone.0017648

**Published:** 2011-03-09

**Authors:** Raúl Teruel, Javier Corral, Virginia Pérez-Andreu, Irene Martínez-Martínez, Vicente Vicente, Constantino Martínez

**Affiliations:** Centro Regional de Hemodonación, University of Murcia, Spain; Oregon State University, United States of America

## Abstract

MicroRNAs (miRNAs) are an abundant class of small non-coding RNAs that are negative regulators in a crescent number of physiological and pathological processes. However, their role in haemostasis, a complex physiological process involving multitude of effectors, is just beginning to be characterized. We evaluated the changes of expression of miRNAs in livers of neonates (day one after birth) and adult mice by microarray and qRT-PCR trying to identify miRNAs that potentially may also be involved in the control of the dramatic change of hepatic haemostatic protein levels associated with this transition. Twenty one out of 41 miRNAs overexpressed in neonate mice have hepatic haemostatic mRNA as potential targets. Six of them identified by two *in silico* algorithms potentially bind the 3′UTR regions of *F7*, *F9*, *F12*, *FXIIIB*, *PLG* and *SERPINC1* mRNA. Interestingly, miR-18a and miR-19b, overexpressed 5.4 and 8.2-fold respectively in neonates, have antithrombin, a key anti-coagulant with strong anti-angiogenic and anti-inflammatory roles, as a potential target. The levels of these two miRNAs inversely correlated with antithrombin mRNA levels during development (miR-19b: R = 0.81; p = 0.03; miR-18a: R = 0.91; p<0.001). These data suggest that miRNAs could be potential modulators of the haemostatic system involved in developmental haemostasis.

## Introduction

MicroRNAs (miRNAs) are an abundant class of small non-coding RNAs of ∼22 nucleotides in length that function as negative gene regulators [1], [2]. In animals, miRNAs are involved in processes such as tissue development and cell differentiation [3], apoptosis [4], in which fine regulation of gene expression in time and space is required for the correct execution of these processes; and in diseases such as cancer [5]. These known functions may represent just a small part of a much bigger scenario; the main known function of miRNAs is the regulation of gene expression at the post-transcriptional level either by protein translation inhibition or mRNA decay [6], [7]. One third of the genes in the human genome are predicted to be miRNAs targets [8] and the continuing discovery of new miRNAs functions suggest that these molecules are implicated in the regulation of almost every physiological processes. Profile studies have already shown that many miRNAs are specifically expressed in certain organs, cell types and developmental stages [3].

To date only two recent studies have demonstrated that key haemostatic proteins, PAI-1 and fibrinogen, may be regulated by miRNA [9], [10], probably just reflecting the tip of the iceberg concerning regulation of haemostasis by miRNA. In this study, we tried to show the potential relevance that miRNAs regulation might have in the whole haemostatic system, by evaluating the differential expression of miRNAs in the liver of mice associated with the extraordinary change in the expression of hepatic haemostatic proteins after birth. Developmental haemostasis refers to the age-related changes in the coagulation system that are most marked during neonatal life and childhood [11]. Actually, the haemostatic system is incompletely developed at birth and matures throughout infancy. Neonates have low levels of the most procoagulant and anticoagulant proteins, although the levels of the factors V, VIII, XIII, and fibrinogen in neonates are similar to adult [12]. The most intriguing aspect of developmental haemostasis is to understand the mechanisms and rationale for such marked age-related changes. Previous studies have confirmed that post-translational modifications in coagulation proteins do occur with age. Moreover, significant differences at the transcriptional levels can also contribute to these differences. However, due to the role of miRNAs in development, these molecules may also contribute directly or indirectly to the dramatic changes in the haemostatic system observed in neonates [2], [12].

## Results

**Figure 1:**
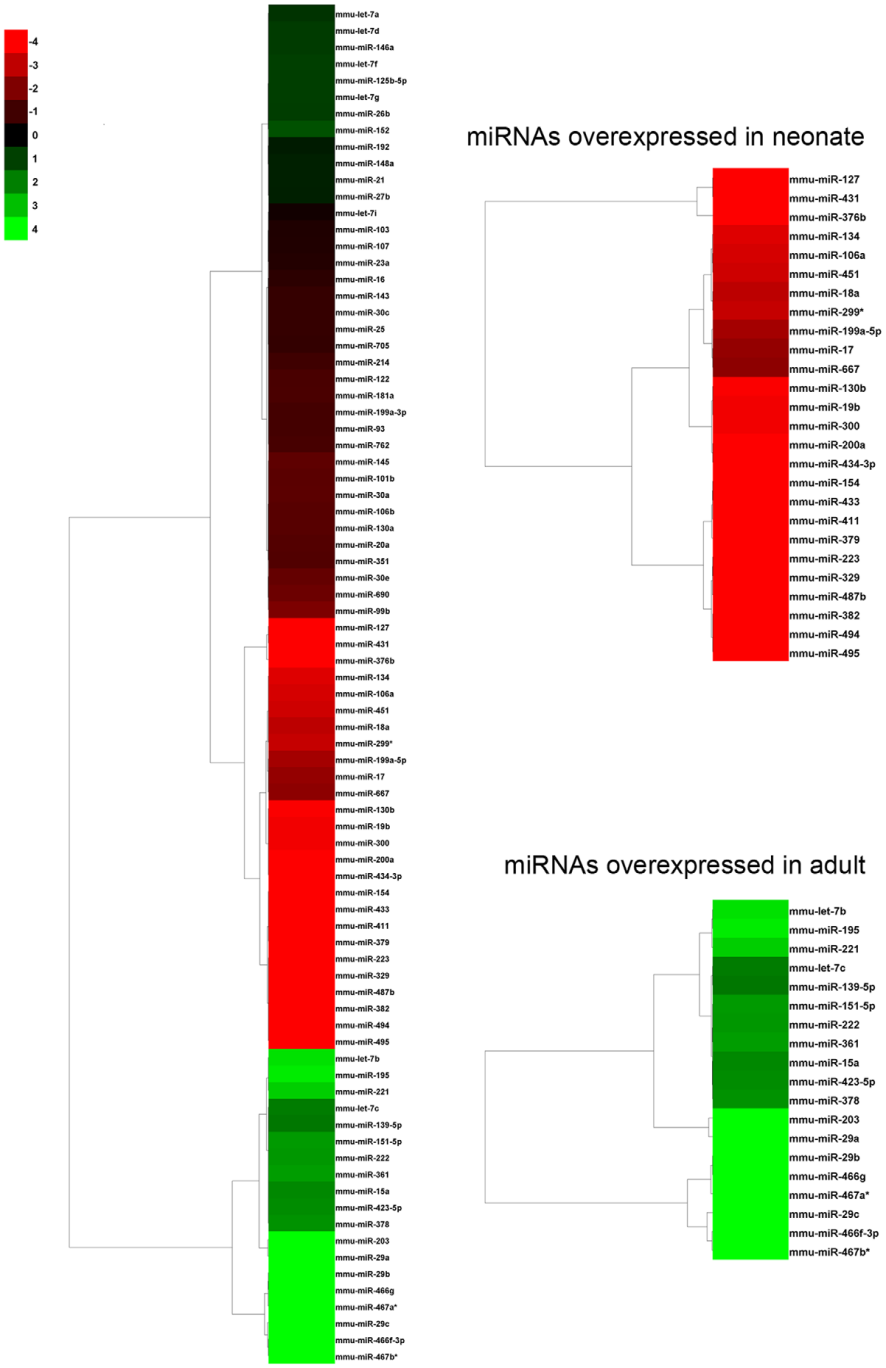
miRNA expression profile. Differentially expressed miRNAs were clustered by Cluster3.0. Log2 scale was employed. In red miRNAs overexpressed in neonate liver; in green miRNAs overexpressed in adult liver.

**Figure 2:**
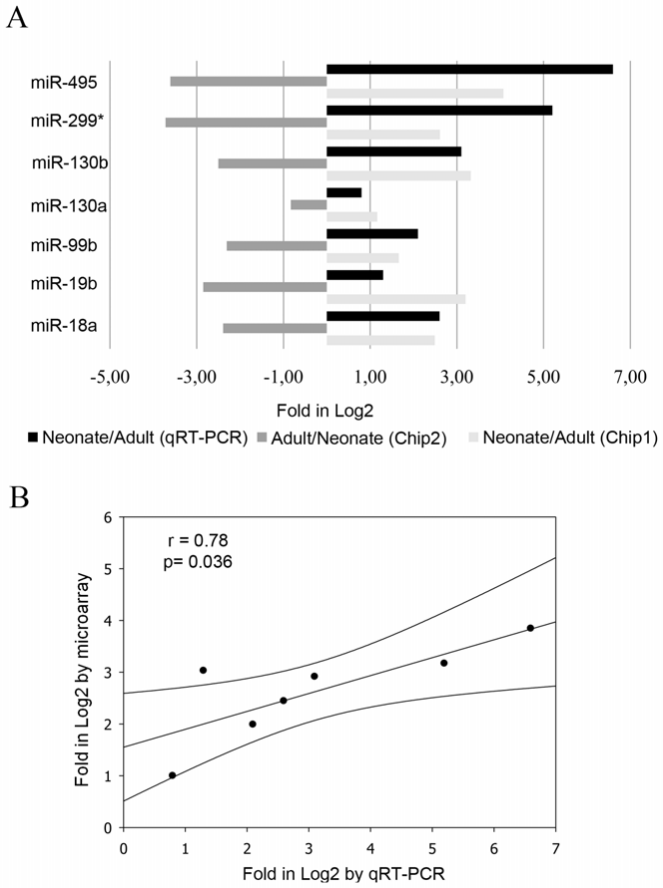
miRNA expression validation. (A) Selected miRNAs were validated by RT-PCR using specific assays. (B) Correlation between data from microarray and RT-PCR for selected miRNAs was performed using lineal regression (SPSS15.0 software). P<0.05 was considered as statistically significant.

**Figure 3:**
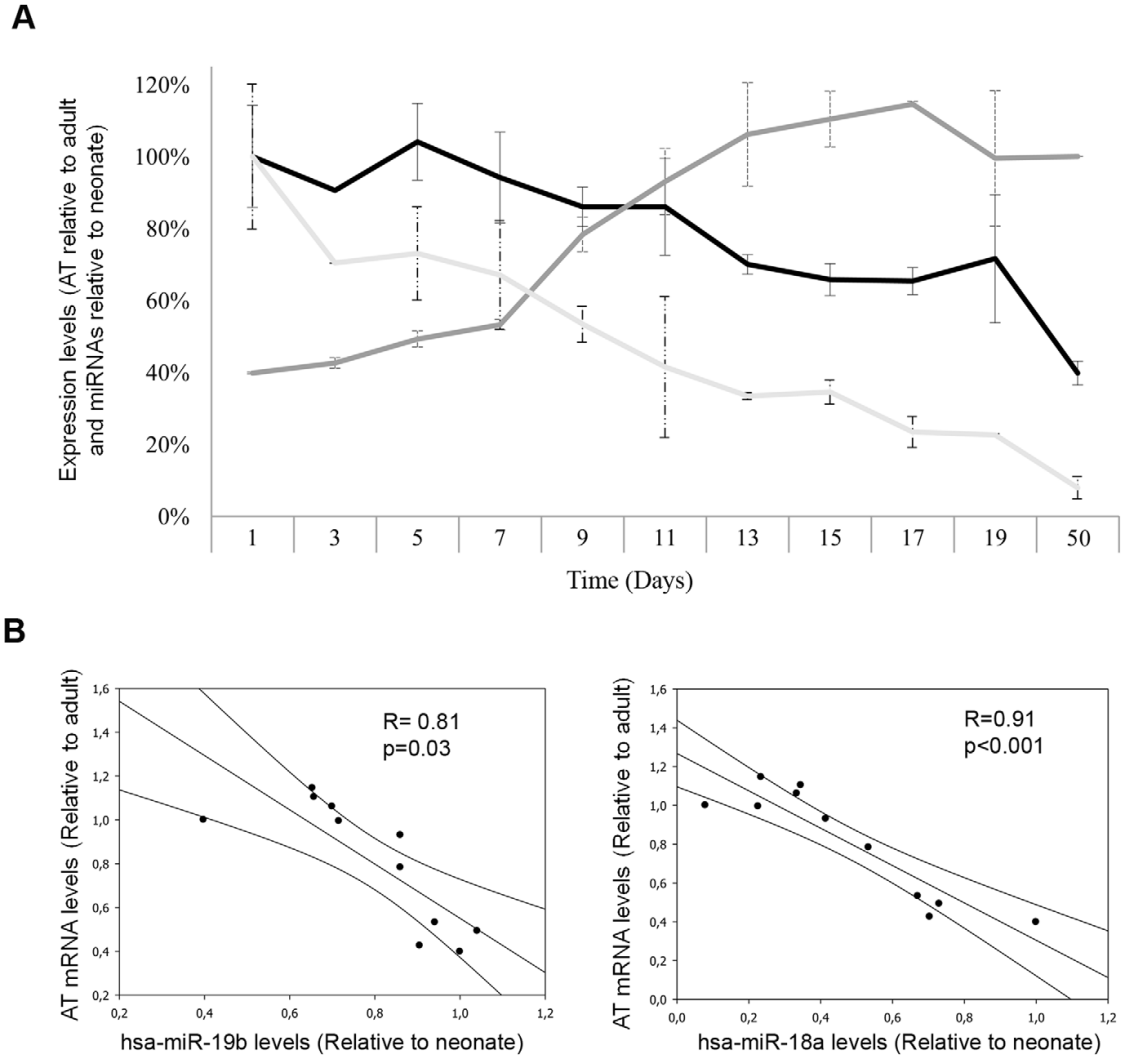
Expression of antithrombin and miRNAs miR-18a and miR-19b during post-natal development in mouse. (A) miRNAs and antithrombin mRNA were measured in liver from mice during postnatal development using cDNA specific assays. RT-PCR was performed in three mice from each age. Data are shown as relative level (% ± SD) taking adults as reference for antithrombin mRNA and +1 day neonates for miRNAs. (B) Expression correlation between miR-19b and miR-20a levels with AT mRNA levels, during post-natal development.

We studied the differential expression pattern of 558 mature miRNAs in liver from an adult and a neonate mouse. As expected, we found that miRNAs expression profiles significantly differed in these two stages. The expression level of 81 miRNAs significantly changed between livers from adult and neonate (Figure 1). We filtered these data by the fold change, and found that 68 out of 81 miRNAs showed at least a two-fold expression change between neonate and adult liver (Table 1). Among these miRNAs, 41 were overexpressed in neonate in comparison with adult and 27 miRNAs were overexpressed in adult when compared to neonate (Table 1). If certain miRNAs were directly involved in the control of the expression of haemostatic proteins, we could find such candidates among the miRNA overexpressed in neonates. Thus, we performed an exhaustive *in silico* search of haemostatic proteins that might be potential targets of the 41 miRNAs overexpressed in neonate mice. Interestingly, 21 out of 41 miRNAs overexpressed in neonate mice, have haemostatic mRNA as potential targets (Table 2). When we focused on those targets predicted by both algorithms, we found 6 miRNA that potentially can bind the 3′UTR regions of mRNA from *F7* (encoding the coagulation factor VII), *F9* (encoding the coagulation factor IX), *F12* (encoding the coagulation factor XII), *FXIIIB* (encoding the coagulation factor XIII, B polypeptide), *PLG* (encoding plasminogen), and *SERPINC1* (encoding the anticoagulant antithrombin) genes. All these proteins are significantly lower in neonates compared with adults in humans although no data are available in mice. Then we validated the expression of these miRNA observed in the microarray in 14 neonate mice (+1 day after bird) and 6 adult mice by qRT-PCR. The results obtained were fully comparable, and the relative fold changes of miRNA expression were similar to those detected by microarrays (r = 0.78, p<0.05) (Figure 2). We were particularly interested to deepen in two miRNAs: miR-18a and miR-19b which are overexpressed 5.4 and 8.2-fold and both have antithrombin as a potential target. Antithrombin is a key anticoagulant that also plays other relevant roles outside the haemostatic system such as strong anti-angiogenic [13] and anti-inflammatory [14] roles. Moreover, the levels of this serpin are naturally reduced in newborns to less than 50% of the levels observed in adults and then increase to approach adult levels by approximately six months of age in humans [15]. First, we confirmed the lower antigenic levels of antithrombin in plasma of neonate mice compared with adults (34±4 *vs.* 86±7, respectively), reaching adult levels at +13 days after birth. Interestingly, when quantifying miR-18a, miR-19b, and antithrombin mRNA during the 19 days after birth, we found an inverse and significant correlation (miR-19b: R = 0.81; p = 0.03; miR-18a: R = 0.91; p<0.001) (Figure 3). We point out that miR-18a and miR-19b are also expressed in human liver where their expression pattern during development is similar to that observed in our study [16].

**Table 1:** Potential haemostatic factor mRNA targeted by miRNAs overexpressed in neonate liver.

miRNA	Fold change	mirGen	Targetscan
**mmu-miR-376b**	85,6	-	Protein C, Factor V
**mmu-miR-154**	26,5	-	Factor IX
**mmu-miR-379**	26,9	Factor VIII	Factor V
**mmu-miR-223**	15,1	**Factor IX** #, **plasminogen** #	Factor VIII, **Factor IX** #, **Plasminogen** #
**mmu-miR-495**	14,3	Factor II	-
**mmu-miR-382**	17,9	Factor XIII (beta subunit), Plasminogen	-
**mmu-miR-200a**	8,4	Factor XIII (beta subunit)	-
**mmu-miR-130b**	7,5	Antithrombin, Factor XIII (beta subunit)	Protein C
**mmu-miR-19b**	8,2	**Antithrombin** #, Factor XIII (beta subunit)	**Antithrombin** #
**mmu-miR-300**	9,2	**Factor XIII (beta subunit)** #	**Factor XIII (beta subunit)** #
**mmu-miR-134**	8,2	**Factor VII** #	**Factor VII** #
mmu-miR-299*	9,0	Factor II, Factor XI	-
**mmu-miR-199a-5p**	4,3	-	Factor XI, Factor XII
**mmu-miR-18a**	5,4	**Antithrombin** #	**Antithrombin** #
**mmu-miR-667**	5,2	Protein S (alpha), Factor VII	-
**mmu-miR-99b**	4,0	Plasminogen, Factor II	-
**mmu-miR-30e**	2,3	-	Factor XI
**mmu-miR-145**	2,8	Plasminogen	-
**mmu-miR-30a**	2,5	-	Factor XI
**mmu-miR-130a**	2,0	Antithrombin, Factor XIII (beta subunit)	Protein C
**mmu-miR-181a**	2,4	Factor VII, Factor X, **Factor XII** #, Protein S (alpha)	**Factor XII** #, FactorXI

# mRNA targeted in both algorithms.

**Table 2:** Potential haemostatic factor mRNA targeted by miRNAs over-expressed in neonate liver.

*miRNA*	*mirGen*	*Targetscan*
¶ **mmu-miR-223**	**Factor IX** *, **Plasminogen** *	Factor VIII, **Factor IX** *, **Plasminogen** *
¶ **mmu-miR-19b**	**Antithrombin** *, Factor XIII (beta subunit)	**Antithrombin** *
¶ **mmu-miR-300**	**Factor XIII (beta subunit)** *	**Factor XIII (beta subunit)** *
¶ **mmu-miR-134**	**Factor VII** *	**Factor VII** *
¶ **mmu-miR-18a**	**Antithrombin** *	**Antithrombin** *
# **mmu-miR-181a**	Factor VII, Factor X, **Factor XII** *, Protein S (alpha)	Factor XI, **Factor XII** *

¶ Overexpressed 4–32-fold in comparison with adult liver,

# overexpressed 2–4-fold in comparison with adult liver.

*mRNA targeted in both algorithms.

## Discussion

Our study has identified 41 miRNAs overexpressed in livers of neonate mice, some of them with potential direct effect on hepatic haemostatic proteins that dramatically change their levels after birth. The inverse correlation observed between miR-18a and miR-19b levels with antithrombin mRNA, one potential target of these miRNAs, suggests that certain miRNAs may be involved in the regulation of selected hepatic haemostatic proteins during development by targeting mRNA coding for these proteins and be in part responsible of the observed decay in neonates [12]. Indeed, Tzur et al. suggested that in humans several biological processes and pathways, in particular blood coagulation, may be regulated during the embryonic period by differentially expressed miRNAs [16]. Beside the potential role of miRNAs in the regulation of haemostatic proteins in developmental haemostasis in liver, these molecules may also be of relevance in adults where the wide inter-individual variability for haemostatic proteins may be potentially responsible of bleeding or thrombotic disorders. In addition, miRNAs may also play a role through indirect mechanisms, by regulating transcriptional factors or post-translational modifications affecting haemostatic elements. Several studies have shown that certain miRNAs may regulate the levels of transcription factors expressed in the liver, potentially involved in the regulation of the transcription of a large panel of hepatic haemostatic mRNAs [17], [18], [19]. Additionally, our results are based on a microarray performed on the expression of 558 miRNAs, the use of novel miRNA databases would certainly add additional miRNA candidates as potential regulators of other hepatic haemostatic proteins not described in our study. Obviously, the main limitation of our study is that it did not demonstrate through *in vitro* experiments the validity of our hypothesis. To note, we looked for a cellular model that endogenously express antithrombin in order to inhibit its expression with oligonucleotide precursors. First, we examined AML12, a murine hepatocyte cell line that unexpectedly, did not express antithrombin. We next move to NIH3T3 cells that do express antithrombin and we performed transfection assays with oligonucleotide precursors and inhibitors (pre-miRs and antagomiRs from Applied Biosystems, Madrid, Spain) of miR-18a and miR-19b to evaluate their effect in the expression of antithrombin. Our results suggest that these miRNAs might not have a direct effect on antithrombin levels in this cell line (data not shown). However, this cellular model had several pitfalls. Particularly, NIH3T3 cells are not a hepatic cell line, and accordingly, cytosolic environment may be completely different from that of hepatocytes. Moreover, the level of expression of antithrombin is very low, and fibroblasts do not express antithrombin in the adulthood. Further studies are required to sustain the potential role of miRNA in the dramatic change of the haemostatic system after birth. Thus, it is necessary to clarify the mechanisms of action of candidate miRNA overexpressed in neonates on the levels of haemostatic proteins. Any potential direct inhibitory effect of miRNAs must be demonstrated under appropriate conditions: i.e. embryonic mouse hepatocytes transfected with miRNA inhibitors or adult mouse hepatocytes transfected with miRNA oligonucleotide precursors. On the other hand, the indirect effect of miRNA on haemostatic protein regulation by targeting transcription factors may also be extremely relevant to evaluate.

In conclusion, these results open new fascinating perspectives in thrombosis and haemostasis by introducing novel elements, miRNAs, as potential regulators of the haemostatic system.

## Methods

### Mouse samples

For microarray assay, we sacrificed one mice of different stage of development: one day after birth mouse (neonate) and fifty days after birth mouse (adult). On the other hand, we used 6 adult mice and 14 neonate mice from at 4 different litters, which did not include the samples used for the microarray assays, to perform miRNA expression validation assay. Finally, we sacrificed 3 mice from different litters, from neonate stage (day +1) to adult stage (day +50) each two days, to perform the quantification of antithrombin mRNA levels and miRNAs miR-18a and miR-19b levels, during post-natal development. In all case we used swiss (ICR CD-1™) mice. Livers were finely dissected and immediately frozen in liquid nitrogen and kept at −80°C until their use. Blood was anticoagulated with citrate and plasma stored at −80°C.

All experimental procedures were performed in accordance with the approved Institutional Animal Care Guidelines from the University of Murcia. The study was approved by the Ethics Committee from Fundación para la Formación e Investigación Sanitarias de la Región de Murcia (04–23/2008).

### RNA Isolation

Total RNA was isolated from frozen liver using Trizol® Reagent (Invitrogen, Carlsbad, CA) following manufacturer's instructions. The RNA concentration and 260/280 ratio were determined by using NanoDrop spectrophotometer (Thermo Scientific, Wilmington, DE) and RNA integrity was verified by lab-on-chip technology using the Experion automated electrophoresis system (Bio-Rad Laboratories, Madrid, Spain).

### MicroRNA microarray

MicroRNAs microarray profiling was performed using total RNA extracted from the liver from one adult mouse (day +50) and one neonate mouse (day +1) using the LC Sciences technology (LC Sciences, Houston, TX). The arrays were designed to detect and quantify miRNA transcripts corresponding to 558 mature miRNAs contained in the Sanger mirBase Release 10.0 (miRMouse 10.0) http://www.sanger.ac.uk/Software/Rfam/mirna/. We used two chips in which RNAs from each sample were labeled either with cy3 or with cy5. The signal values were derived by background subtraction and normalization. A transcript to be listed as detectable must meet at least two conditions: signal intensity higher than 3×(background SD) and spot CV<0.5. CV is calculated by (SD)/(signal intensity). When repeating probes were present on an array, a transcript was listed as detectable only if the signals from at least 50% of the repeating probes were above detection level. Signals were listed in median signal values of repeating probes of p-value<0.01. Median values were used to minimize the effect of occasional “non-uniform spots” that may have signal values deviate from average signal values but have p-values<0.01.

All differentially expressed transcripts with p-value<0.01, were presented in log2 scale, positive log2 value indicates an upper regulation and a negative log2 value indicates a down regulation.

### Validation by qRT-PCR

The RT reaction was performed using 200 ng of total RNA for each sample according to the manufacturer instructions (SuperScript First Strand, Invitrogen, Madrid, Spain). The Real Time reactions were performed using Taqman Gene Expression Assay on a LC480 Real Time PCR (Roche, Madrid, Spain). For mature miRNA quantification one set of primers and a probe were chosen from Applied Biosystems. Expression analysis was performed in triplicates for each sample. Expression of U6 snRNA was used as endogenous reference control. The fold difference for each sample was obtained using the 2^−ΔΔCt^ method.

### Target analysis

*In silico* search of potential miRNA targets was performed in the following miRNA-target prediction algorithms : TargetScan (release 5.1, http://www.targetscan.org) [8], mirGen (http://www.diana.pcbi.upenn.edu/miRGen) [20].

### Statistical analysis

Lineal regression was performed by Statistical Package for Social Science (version 15.0; SPSS, Chicago, IL, USA). Data are presented as mean ± standard deviation. Statistical significance was taken as P<0.05.
